# The ECG spoke first: bifascicular block in acute anterior myocardial infarction

**DOI:** 10.1093/ehjcr/ytag201

**Published:** 2026-03-16

**Authors:** Sofia Esteves, Catarina Santos Gregório, Gustavo Lima da Silva

**Affiliations:** Department of Cardiology, Unidade Local de Saúde de Santa Maria, CAML, CCUL@RISE, Faculdade de Medicina, Universidade de Lisboa, Avenida Professor Egas Moniz, MB, Lisboa, Portugal 1649-035, Lisboa; Department of Cardiology, Unidade Local de Saúde de Santa Maria, CAML, CCUL@RISE, Faculdade de Medicina, Universidade de Lisboa, Avenida Professor Egas Moniz, MB, Lisboa, Portugal 1649-035, Lisboa; Department of Cardiology, Unidade Local de Saúde de Santa Maria, CAML, CCUL@RISE, Faculdade de Medicina, Universidade de Lisboa, Avenida Professor Egas Moniz, MB, Lisboa, Portugal 1649-035, Lisboa

## Clinical presentation

**Figure 1.**
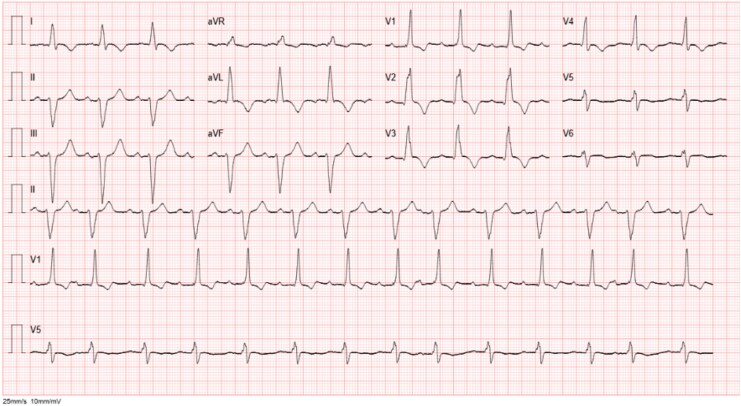
ECG at presentation.

An 88-year-old man presented to the emergency department with acute-onset chest pain and nausea. His past medical history was notable for pre-existing right bundle branch block (see Supplementary material online, *Figure S1*), chronic kidney disease, and follicular lymphoma. Transthoracic echocardiography revealed severely reduced left ventricular systolic function (left ventricular ejection fraction of 24% with the Simpson biplane method), secondary to apical akinesia and hypokinesia of the anteroseptal and anterior walls. The initial ECG is shown in *Figure 1*.

Shortly after presentation, the patient developed complete atrioventricular block with cardiac arrest (see Supplementary material online, *Figure S2*) requiring emergent transfemoral temporary pacing. Coronary angiography revealed acute occlusion of the proximal left anterior descending artery, consistent with an extensive anterior myocardial infarction. Transthoracic echocardiography showed severely reduced left ventricular systolic function due to apical akinesia and hypokinesia of the anterior and anteroseptal walls.

Following coronary reperfusion, conduction through the posterior fascicle recovered, while the left anterior fascicular block persisted, indicating incomplete recovery and permanent injury of the His–Purkinje system (see Supplementary material online, *Figure S3*).

## Question 1

**Which interpretation of the admission electrocardiogram is most appropriate?**

- Incidental age-related conduction disease with no immediate clinical implications.
- New ischaemic involvement of the anteroseptal conduction system due to proximal left anterior descending artery occlusion.
- Reversible metabolic conduction abnormality related to electrolyte imbalance.
- Acute inferior myocardial infarction with predominant atrioventricular nodal involvement.
- Acute pericarditis with secondary conduction slowing.

## Question 2

**Which coronary artery lesion most plausibly explains the ECG findings?**

- Distal right coronary artery occlusion affecting the atrioventricular node
- Mid–left circumflex artery occlusion causing lateral wall ischaemia
- Proximal left anterior descending artery occlusion involving septal perforators supplying the His–Purkinje system
- First diagonal branch occlusion with isolated anterior wall involvement
- Acute left main coronary artery occlusion with diffuse subendocardial ischaemia

## Question 3

**What is the most appropriate immediate management strategy?**

- ECG monitoring, cardiac biomarkers, and anti-ischaemic, anticoagulation, and antithrombotic therapy.
- Initiation of antiarrhythmic therapy to prevent progression of conduction disease.
- Urgent coronary reperfusion with readiness for temporary pacing due to high risk of complete atrioventricular block.
- Permanent pacemaker implantation prior to coronary angiography.
- Conservative medical management given the advanced patient age.

### Answer and discussion

**Correct answers:**

Question 1: **B**

Question 2: **C**

Question 3: **C**

#### Explanation of answers

Explanations for correct answers

Question 1—Correct answer: B. The combination of acute chest pain and a new fascicular block in a patient with pre-existing right bundle branch block strongly suggests acute ischaemic injury of the anteroseptal His–Purkinje system. This region is supplied by septal branches of the proximal left anterior descending artery. According to ESC guidelines on acute coronary syndromes,^1,2^ new conduction abnormalities in the setting of suspected myocardial infarction identify a high-risk presentation and should raise concern for extensive anterior infarction with potential progression to complete atrioventricular block.

Question 2—Correct answer: C. The proximal left anterior descending artery gives rise to septal perforators that supply the anteroseptal myocardium and the His–Purkinje conduction system. Occlusion at this level can therefore produce both extensive anterior wall ischaemia and infranodal conduction disturbances, including bifascicular block and complete atrioventricular block.^3^ This anatomical–electrocardiographic correlation is well described in patients with anterior myocardial infarction and is emphasized in ESC documents addressing conduction disorders in acute ischaemia.^4^

Question 3—Correct answer: C. Recognition of this ECG pattern identifies a high-risk acute coronary syndrome requiring immediate invasive management. ESC guidelines recommend urgent coronary angiography and reperfusion in patients with ongoing ischaemia and high-risk features, including new conduction disturbances. Given the high likelihood of progression to complete atrioventricular block in anterior infarction with bifascicular block, continuous monitoring and readiness for temporary pacing are essential components of early management.

## Teaching point

In patients with pre-existing right bundle branch block, the development of a new fascicular block during acute chest pain strongly suggests proximal left anterior descending artery occlusion and identifies patients at high risk for conduction-related cardiac arrest.

## Supplementary Material

ytag201_Supplementary_Data

## Data Availability

No new data were generated or analysed in support of this research.

## References

[1] Collet J-P, Thiele H, Barbato E, Barthélémy O, Bauersachs J, Bahtt D, et al 2020 ESC guidelines for the management of acute coronary syndromes in patients presenting without persistent ST-segment elevation. Eur Heart J 2021;42:1289–1367. 32860058 10.1093/eurheartj/ehaa575

[2] Ibanez B, James S, Agewall S, Antunes M, Bucciarelli-Ducci C, Bueno H, et al 2017 ESC guidelines for the management of acute myocardial infarction in patients presenting with ST-segment elevation. Eur Heart J 2018;39:119–177. 28886621 10.1093/eurheartj/ehx393

[3] Glikson M, Nielsen JC, Kronborg MB, Michowitz Y, Auricchio A, Barbash I, et al 2021 ESC guidelines on cardiac pacing and cardiac resynchronization therapy. Eur Heart J 2021;42:3427–3520. 34586378 10.1093/eurheartj/ehab699

[4] Surawicz B, Knilans TK. Chou’s Electrocardiography in Clinical Practice. 6th ed. Philadelphia: Elsevier Saunders; 2008.

