# Supplementary figures and images for: The ECG spoke first: bifascicular block in acute anterior myocardial infarction
Source: Eur Heart J Case Rep. 2026 Mar 16;10(3):ytag201. doi: 10.1093/ehjcr/ytag201 (PMC13037466; doi:10.1093/ehjcr/ytag201)

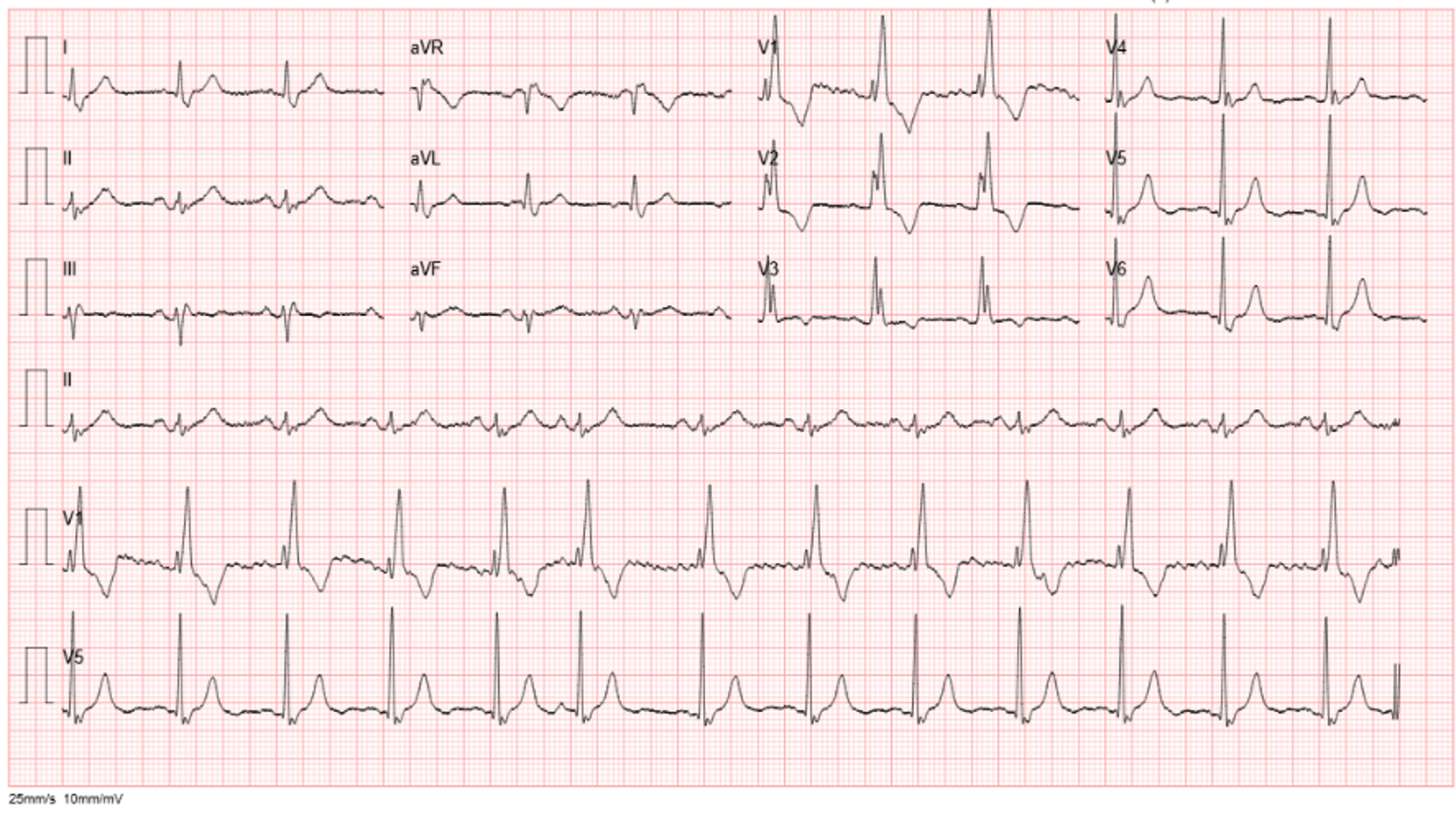

Supplement: ytag201_Supplementary_Data [file ytag201_supplementary_data.zip › Supplementary Figure 1.tiff]

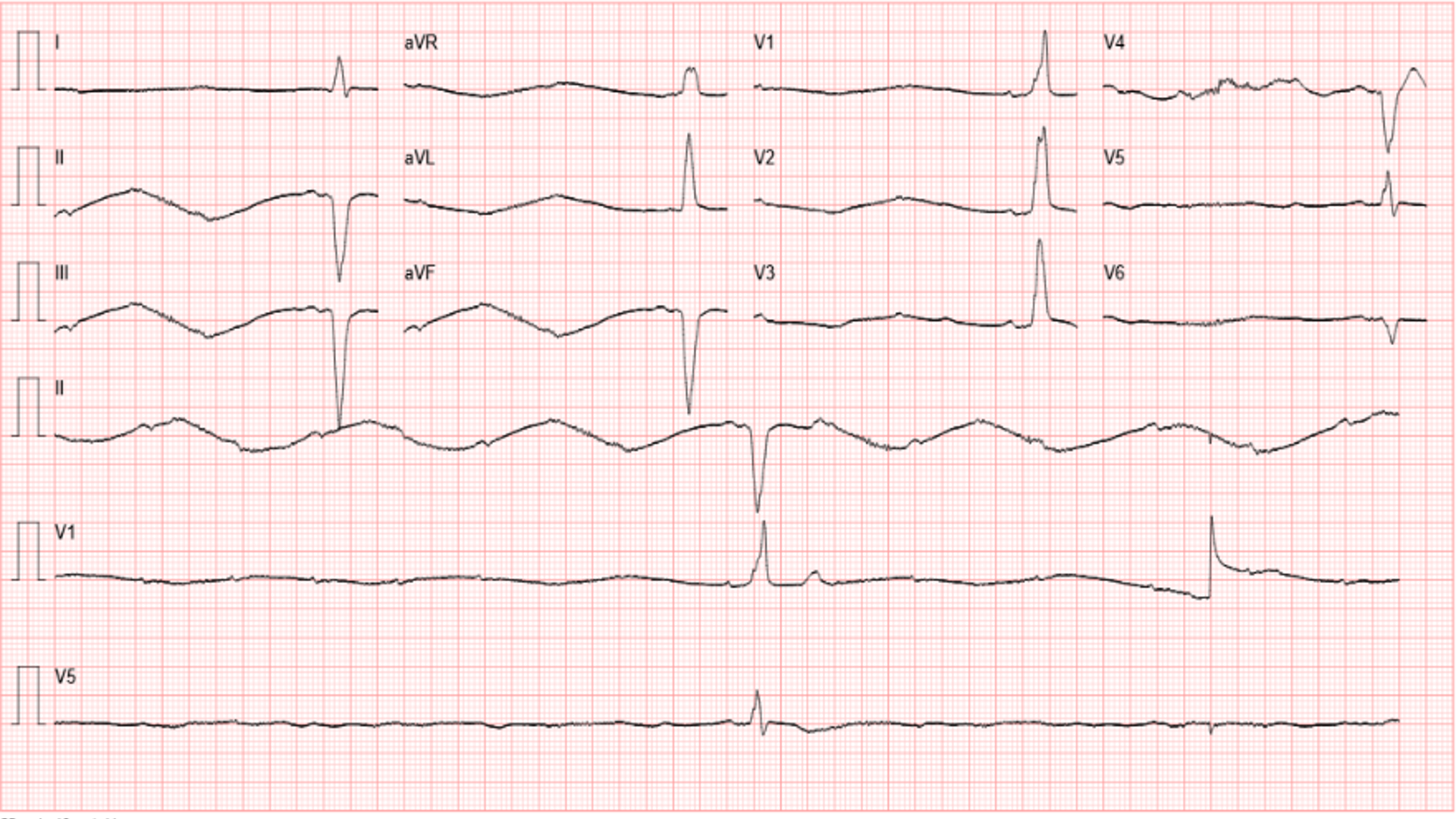

Supplement: ytag201_Supplementary_Data [file ytag201_supplementary_data.zip › Supplementary Figure 2.tiff]

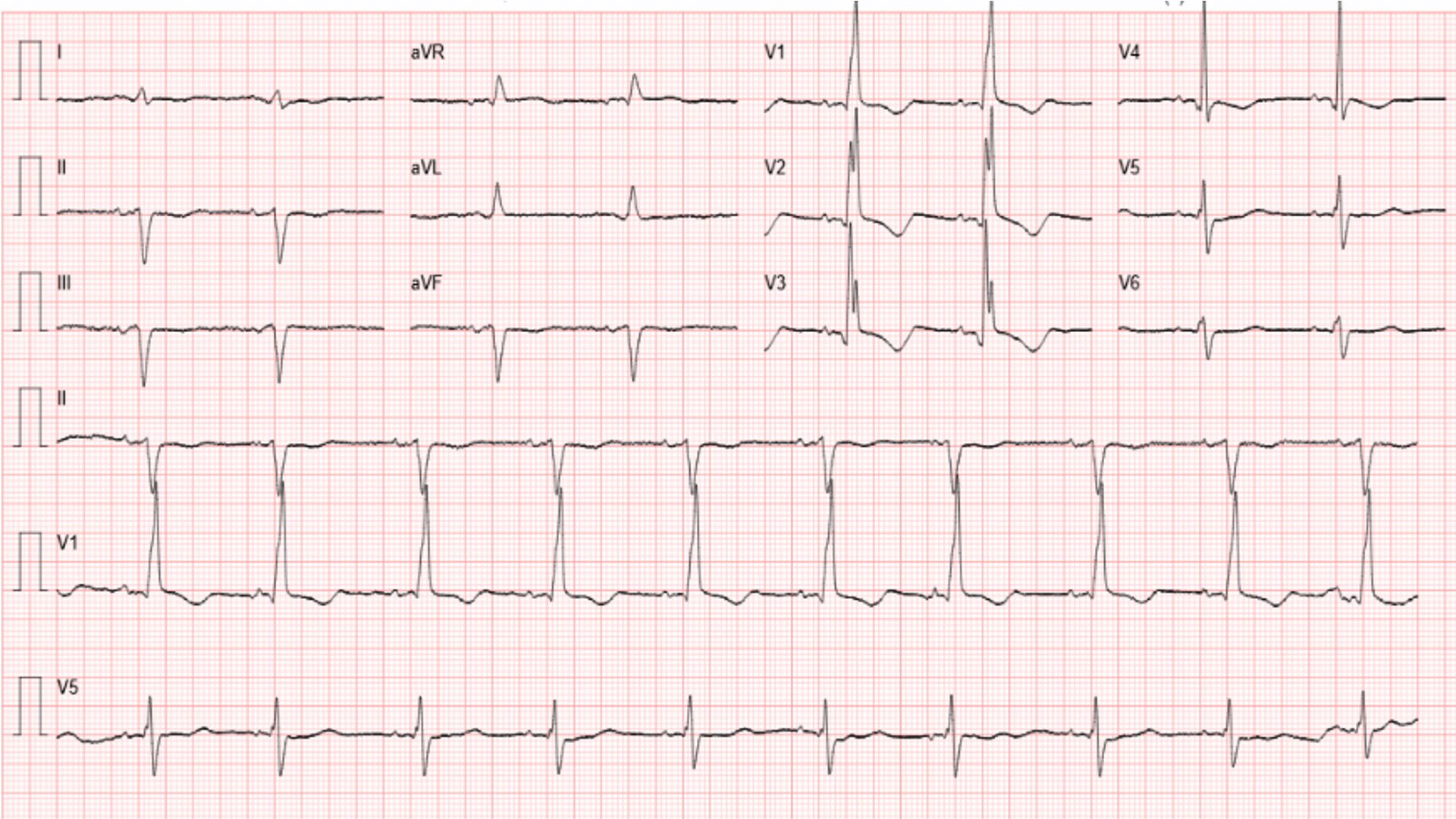

Supplement: ytag201_Supplementary_Data [file ytag201_supplementary_data.zip › Supplementary Figure 3.tiff]
